# Estimating the Volume of Unknown Inclusions in an Electrically Conducting Body with Voltage Measurements

**DOI:** 10.3390/s19030637

**Published:** 2019-02-02

**Authors:** Antonio Affanni, Ruben Specogna, Francesco Trevisan

**Affiliations:** Polytechnical Department of Engineering and Architecture, University of Udine, Via delle Scienze 206, 33100 Udine, Italy; ruben.specogna@uniud.it (R.S.); francesco.trevisan@uniud.it (F.T.)

**Keywords:** Electrical Impedance Tomography, volume estimation, inclusion volume, four wires measurement

## Abstract

We propose a novel technique to estimate the total volume of unknown insulating inclusions in an electrically conducting body from voltage measurements. Unlike conventional Electrical Impedance Tomography (EIT) systems that usually exhibit low spatial resolution and accuracy, the proposed device is composed of a pair of driving electrodes which, supplied with a known sinusoidal voltage, create a current density field inside a region of interest. The electrodes are designed to generate a current density field in the region of interest that is uniform, to a good approximation, when the inclusions are not present. A set of electrodes with a polygonal geometry is used for four-wires resistance measurements. The proposed technique has been tested designing a low cost prototype, where all electrodes are on the bottom of the conducting body, showing good performances. Such a device may be used to monitor the volume of biological cells inside cell culture dishes or the volume of blood clots in micro-channels in lab-on-a-chip biosensors.

## 1. Introduction

There is an increasing interest in the measurement of the volume of inclusions growing inside an investigation area, with lots of applications in engineering [1,2,3]; in particular, in this paper we focus on its application in biomedical engineering and lab-on-a-chip biosensors. An appealing method to implement the volume estimation is to use the electrical impedance or voltage measurements which proved to be faster and more cost effective than techniques based on confocal microscopy.

Unlike conventional EIT systems that exhibit low spatial resolution and accuracy [4,5,6,7,8,9,10,11,12,13], we are concerned here only in the estimation of the total volume of unknown insulating inclusions in an electrically conducting body from voltage measurements. In other words, we are not interested in the position and shape of the inclusions and we assume that the electrical conductivity of the inclusions is negligible with respect to the conductivity of the conducting body.

The considered problem is typical in biomedical engineering when, for example, an automated technique to monitor the cell growth in a dish culture is needed. Another application in the medical field is the real time estimation of the blood clots’ volume in flowing blood for studying the thrombotic process in humans [14,15,16]. Either the cell membranes or the blood clot can be considered as insulating inclusions in the hundreds of kilohertz range of frequencies. For applications inside lab-on-a-chip biosensors, like the thrombotic profiling, the physical feasibility of the device inside openable micro-channels is of particular interest [17].

In mathematical literature, there are few papers containing the derivation of theoretical bounds for the inclusion volume from one measurement taken from a pair of electrodes [18,19,20]. While these bounds are very important from the theoretical point of view, in practice they are not very useful mainly because these bounds depend on unknown parameters that render the bounds not computable. In [21], a method based on simulation to obtain the previously introduced theoretical bounds is proposed. The results in [21] show that the obtained bounds are not very tight and therefore do not allow an accurate evaluation of the volume of the inclusion.

The solution adopted in this paper is strongly inspired by the technique proposed in [22,23,24,25]. The appealing feature of this technique is that no forward or inverse numerical solver is needed to compute the volume of the inclusion, allowing its real time estimation. The method determines the volume of the inclusion by applying a predefined pattern of injection currents on a number of electrodes placed in the lateral surface of a cylindrical container. Then, the voltages on these electrodes are sampled with two wire measurements. Finally, the size of the volume is simply estimated by summing for all electrodes the product of the injected current and the voltage readout.

We extend the technique in [25] in various ways. First of all, to estimate the inclusion volume in a three-dimensional container that represents the conductive body, we place all the electrodes only on the bottom wall of the cylindrical container. This planar configuration of electrodes enables the determination of the volume of inclusions flowing in microchannels of lab-on-a-chip devices with low cost prototyping; it would be more expensive to place the electrodes on the lateral walls of the microchannel. Moreover, we propose a technique to avoid the injection of a predefined pattern of currents in the electrodes that requires one independent current source for each electrode and a precise calibration of the value of each current. We avoid this complication by introducing a pair of driving electrodes in which a single current is injected [26]. Voltages are sampled on another set of electrodes disposed on a circle in the bottom of the container in such a way that voltages are measured with a four-wires technique. This has also the advantage of avoiding the so-called double layer at the electrode-fluid interface and therefore does not require the use of the complete model for electrodes [21]. The estimation of the inclusions volume is hence obtained using a formula derived from a simple power balance in the region of interest.

The physical realization of the prototype shows good performances in terms of linearity of the estimated volume with respect to the volume of the inclusions. Its real time operation and the limited cost render it appealing for the estimation of the total volume of inclusions for various practical applications.

The paper is structured as follows. Section 2 describes the sensor, in particular the geometric shape of the driving and the sensing electrodes, and the signal conditioning circuitry. Moreover, the theory behind its operation is explained by using the electrical power balance in the region of interest. In Section 3, for the sake of comparison only, a commercial simulation software (COMSOL MultiPhysics) is used to reproduce the behavior of the sensor. In Section 4, the performances of the sensor are compared to the ones provided by the numerical simulation. Finally, in Section 5, the conclusions are drawn.

## 2. Material and Methods

### 2.1. Sensor Description

**Figure 1:**
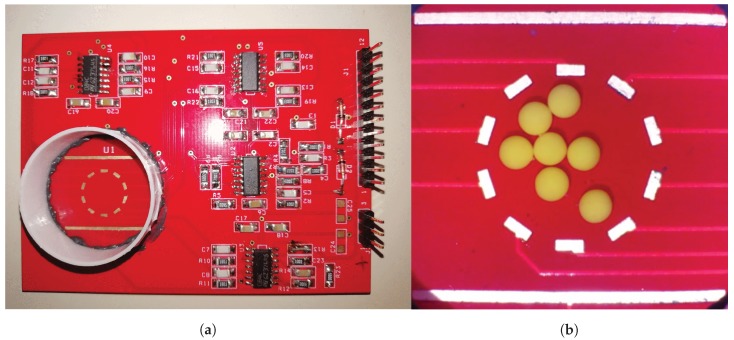
(**a**) overview of the printed circuit board (PCB) with circuitry and sensor (bottom left), and (**b**) zoom of the sensing element with some defects.

The developed sensor and conditioning circuitry have been integrated in a single Printed Circuit Board (PCB) with gold plated electrodes, as shown in Figure 1.

The sensing element (shown in Figure 1b) is formed by: a couple of outer, parallel, wide driving electrodes which are responsible for supplying the sensor providing a current density from the top to the bottom of the sensor; an inner, polygonal set of reading electrodes which perform the readout of the potentials in the investigation area. In the present solution, the polygonal shape is a decagon having a width and height of 10 mm. The driving electrodes are 18 mm wide and 600 µm high, spaced 16 mm apart. The reading electrodes are 1.6 mm wide and 600 µm high posed as a decagon centered with respect to driving electrodes. The sensor forms the bottom wall of a circular reservoir having 25 mm diameter.

**Figure 2:**
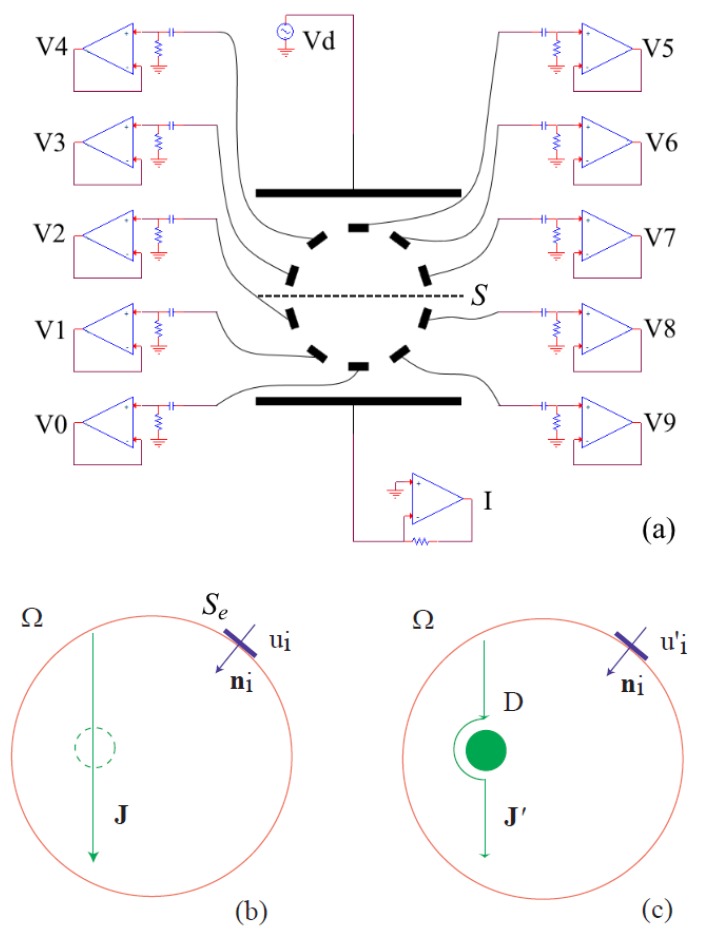
(**a**) Schematic of the board, where potentials $V_{i}$ of the sensing electrodes with i=0,…,9 are shown; moreover, the pair of outer current electrodes have applied voltage Vd and current *I*. (**b**) Current conduction in domain Ω without insulating domain. (**c**) Current conduction in Ω−D, where *D* is the insulating domain.

The schematic of the conditioning circuitry is shown in Figure 2a. A sinusoidal voltage Vd having amplitude 100 mV and frequency 100 kHz is generated by a Wien bridge oscillator with amplitude control; the characterization of the oscillator evidenced a total harmonic distortion (THD) lower than 2% and a stability in the order of 0.3%. The amplitude of Vd has been chosen to avoid red-ox reactions at the driving electrodes (which would yield to electrodes dissolution), while the frequency of Vd has been chosen in order to minimize the double layer effect due to metal-electrolyte interface. Regarding the amplitude in fact, we chose |Vd| = 100 mV, one order of magnitude lower with respect to the half-cell standard potential of gold (which is 1.4 V) to assure that no red-ox reaction takes place, thus avoiding the damage of the current electrodes. The frequency of Vd has been chosen in order to minimize the double layer effect; when a metal transfers charge to an electrolyte, there is the formation of a narrow metal-electrolyte interface, named double layer, which behaves as a constant phase element (CPE). Its impedance is $Z_{CPE}\left(\omega \right)={\left({\left(j\omega \right)}^{\phi }CPE\right)}^{-1}$ and behaves partially as a resistor (if ϕ=0) and partially as a capacitor (if ϕ=1); this leads to a phase shift from voltage to current whose value is −ϕπ/2. In previous works, we demonstrated that if the frequency is ≥100 kHz in the present application the double layer is negligible and current is in-phase with voltages. From the experimental characterization, the phase lag of current resulted lower than 1°; for this reason, in Section 2.3 we safely assume the measured impedance to be a resistance in order to obtain the volume information.

The lower driving electrode is connected to a current-to-voltage converter; its potential is fixed to virtual ground and the current *I* is converted into a voltage by means of a resistor. In the case of the present board, the resistor has been set to 330 Ω in order to obtain that the expected gain of the converter multiplied by the operating frequency results one decade lower than the gain-bandwidth product of the operational amplifier. The characterization of the current to voltage converter provided an uncertainty of conversion in the order of 1%. Each voltage electrode is connected, through a passive first order high-pass filter, to a buffer; the passive filter has input impedance 1.8 MΩ and cut-off frequency at 4 kHz. The aim of the filter is to remove the undesired DC component due to the electrolyte and the power line noise at 50 Hz. The buffer decouples the sensor voltages from the input impedance of the Data Acquisition (DAQ) board connected to the output of the circuit. In this way we minimize the load error during acquisition.

### 2.2. Data Acquisition

**Figure 3:**
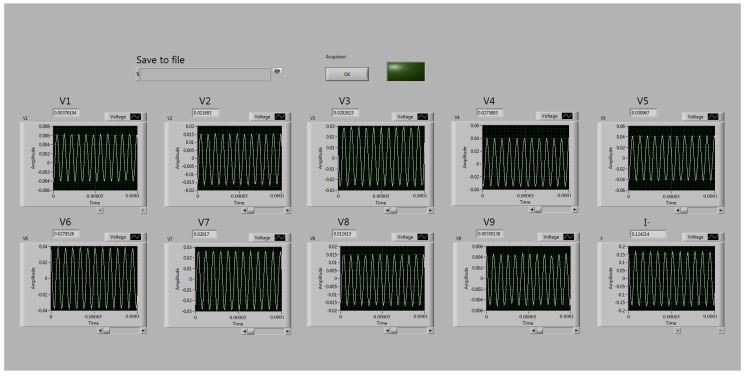
Control panel, developed in LabView, to control the sensor board.

A 16 bits DAQ board is connected to the circuit (National Instruments NI-USB 6353); the DAQ has been set to acquire, at rate 1 MSa/s, a number of 3000 samples (corresponding to 300 periods of the driving voltage) of each differential voltage $u_{i}=V_{i}-V_{0}$, where $V_{i}$ is the potential of each electrode surface $S_{i}$ and $V_{0}$ is the potential of the lower electrode, chosen as reference, Figure 2a. The current signal *I* flowing through the outer electrodes is acquired as a single ended trace referred to ground. A dedicated control panel, shown in Figure 3, developed in LabView, acquires the data of the driving current and of $u_{i}$. During a measurement cycle, the DAQ reads the nine differential voltages ($u_{i}$) and the current signal (*I*). The measurement time for each channel is 3 ms, thus the acquisition time is 30 ms. After acquisition, the control panel calculates the AC-coupled RMS value of each quantity and saves the results into a text file. The volume of the defects is then computed with a Matlab script which calculates the quantity ΔR(D) as described in the next subsection.

### 2.3. Modelling and Volume Reconstruction Formula

Since (from measurements) voltages $u_{i}$ and current *I* are in phase sinusoidal signals, a stationary current conduction model is assumed in the domain of interest Ω, Figure 2b,c. At first, a reference configuration is considered in Ω without any insulating domain *D*, Figure 2b. We denote with *I* the current in this case, yielding a uniform current density vector J in Ω of amplitude

(1) $$J=\frac{I}{S}$$

where *S* denotes the effective cross-section area located between the current electrodes; its precise geometrical extent is not crucial, since *S* plays the role of an instrumental constant. For example, dashed line in Figure 2a represents the trace of a possible surface *S*. The electric power $P_{in}$ entering Ω from its boundary ∂Ω, can be expressed as

(2) $$P_{in}=\sum_{1}^{9}u_{k}i_{k},$$

where the current $i_{k}$ on ∂Ω is

(3) $$i_{k}=J\cdot n_{k}S_{e}=JS_{e}\cos\theta_{k},$$

where $S_{e}$ is the effective area of the electrodes and $\theta_{k}$ is the angle between J and the normal $n_{k}$ to $S_{e}$, with k=1,…,9. Of course $P_{in}$ underestimates the actual electrical power flowing through ∂Ω, due to the discrete distribution of the electrodes on ∂Ω. By substituting (3) in (2), we obtain

(4) $$P_{in}=JS_{e}\sum_{1}^{9}u_{k}\cos\theta_{k}$$

Now, we apply the following approximate power balance in Ω

(5) $$P_{in}=R\left(\Omega ,\rho ,J\right)I^{2},$$

where the functional

(6) $$R\left(\Omega ,\rho ,J\right)=\frac{1}{I^{2}}\int_{\Omega }\rho J^{2}d\Omega$$

is the equivalent resistance of Ω, ρ being the resistivity of the medium filling Ω. By combining (1), (4) and (5), we obtain

(7) $$R\left(\Omega ,\rho ,J\right)=\frac{S_{e}}{S}\sum_{1}^{9}R_{k}\cos\theta_{k},$$

where

(8) $$R_{k}=\frac{u_{k}}{I}.$$

Expression (7) yields an estimate of R(Ω,ρ,J) from voltages on ∂Ω and the current *I*; the quantities $R_{k}$ are deduced directly from resistance measurement at the specified frequency. Moreover, the ratio $S/S_{e}$ plays the role of instrumental constant; the extent of *S* and $S_{e}$ can be estimated by means of numerical simulations.

Next, we introduce in Ω a number of insulating subregions whose union will be denoted with *D*, Figure 2c; thus, the new conducting domain of resistivity ρ becomes Ω−D, and the corresponding resistance functional becomes

(9) $$R^{\prime }\left(\Omega -D,\rho ,J^{\prime }\right)=\frac{1}{I^{\prime }{}^{2}}\int_{\Omega -D}\rho J^{\prime }{}^{2}d\Omega ,$$

where $I^{\prime }$ is the current flowing through the outer electrodes due to the presence of the insulating domain *D*.

Applying a power balance to Ω−D similar to (7), we obtain an estimate of the equivalent resistance of Ω−D from voltages $u_{k}^{\prime }$ on ∂Ω

(10) $$R^{\prime }\left(\Omega -D,\rho ,J^{\prime }\right)=\frac{S_{e}}{S}\sum_{1}^{9}R_{k}^{\prime }\cos\theta_{k},$$

where

(11) $$R_{k}^{\prime }=\frac{u_{k}^{\prime }}{I^{\prime }}.$$

Again, the quantities $R_{k}^{\prime }$ are deduced directly from resistance measurement at the specified frequency.

Finally, from (7) and (10), we compute the quantity

(12) $$\Delta R\left(D\right)=R^{\prime }\left(\Omega -D,\rho ,J^{\prime }\right)-R\left(\Omega ,\rho ,J\right)=\frac{S_{e}}{S}\sum_{1}^{9}\left(R_{k}^{\prime }-R_{k}\right)\cos\theta_{k},$$

which is proportional to volume of *D* since the resistivity of the filling conductive medium ρ is an invariant.

## 3. FEM Simulations

The 3D geometry has been simulated using Comsol Multiphysics with a stationary conduction definition, since all the signals are in-phase. On the circumference that delimits the region we set the boundary condition of insulation, i.e., what happens in experimental setup having a plastic reservoir. We set the upper electrode at potential 100 mV and the lower electrode to ground; we evaluate $V_{i}$ on the voltage electrodes and *I* as the surface integral of current densities on the boundaries of the upper electrode. The subdomain of water has been set 3 mm high (as in experimental conditions) and its conductivity has been iteratively trimmed to match the current measured in the experiments with no defects in the investigation area. Inside the decagon, we have posed 18 spheres having 1.5 mm diameter leaning on the electrodes plane; their conductivities can assume the saline water value (in the order of 4 S/m) or the glass value (in the order of 10 ^−7^ S/m) depending on the simulation setup.

The meshed geometry consists of 20,577 s order tetrahedra. A simulation script performed 10 series of simulations with random permutation of the spheres conductivities; each series is composed by 18 simulations (for a total amount of 180 simulations), where all the spheres are, progressively one by one, switched from water to glass. In this way, we simulate (with the same meshed geometry) 10 different combinations of possible linear growths of the spheres volume in the investigation area.

**Figure 4:**
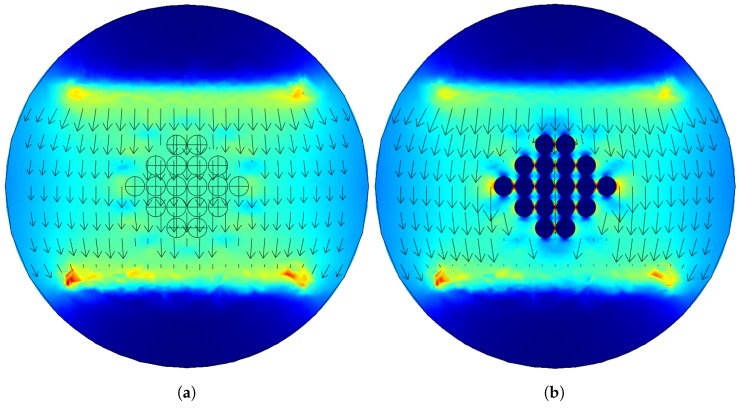
Current density vector simulated with Comsol Multiphysics; arrows represent the current density direction and the surface color represents the magnitude. (**a**) When all the spheres are supposed to be water, (**b**) when all the spheres are supposed to be glass.

Figure 4a shows the simulated current density when all the spheres have the same conductivity of water, while Figure 4b shows the current density distribution when all the spheres have conductivity of glass; it is evident in Figure 4b that the current density avoids the glass insulating spheres, while no discontinuity is manifested in Figure 4a.

## 4. Experimental Results

**Figure 5:**
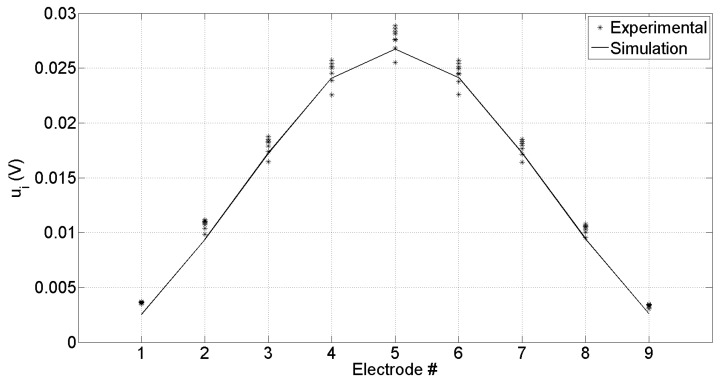
Differential voltage at ith electrode $u_{i}=V_{i}-V_{0}$; line represents the numerical simulation, markers represent experimental results on 10 different measurements without defects in the sensor.

In this section, we show the experimental results obtained from measurements and we compare them to FEM simulations. The reservoir of the sensor has been filled with 1.5 × 10^−6^ m^3^ (corresponding to 3 mm height) saline solution and we performed 10 measurement series to evaluate the linearity of the overall system. Each measurement series started from the acquisition of the voltages and current with no defects in the reservoir; then, progressively one by one, 18 defects (glass spheres with 1.5 mm diameter) have been added into the solution and, for each additional defect, we measured the respective voltages and current. Figure 5 shows the differential potentials at each electrode $u_{i}$ measured without defects in the decagon; markers represent the acquired experimental data, while the line represents the simulation results.

**Figure 6:**
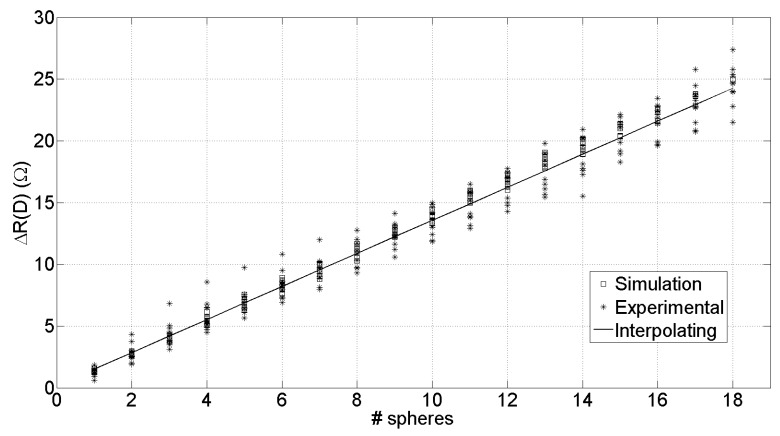
Evaluation of the quantity ΔR(D) over 10 series of experiments: (∗) Simulation results, (□) Experimental results, (—) least squares fitting.

Figure 6 shows the trend of ΔR(D) in (12) for experimental results (∗), simulations (□), and its least squares linear regression (—). As it can be seen, depending on the sequence of insertion of the spheres in the investigation area, there is a small deviation from linearity both in experimental and in simulations.

For this reason, we are interested in evaluating the non-linearity of the sensor and comparing it to the non-linearity manifested in simulations. Evaluating the difference between data and least squares linear regression, we obtain the punctual linearity error of each jth volume acquisition ($v_{jk}$) obtained from the kth series with respect to the least squares linear interpolation. The linearity error thus is evaluated as the difference between linear regression and the mean value $\bar{v_{j}}$ of each point among the 10 series of measurements; the uncertainty on the linearity error $u{\left(Lin\right)}_{j}$ is thus represented by the standard deviation of the sample means:

(13) $$u{\left(Lin\right)}_{j}=\sqrt{\frac{\sum_{k=1}^{10}{\left(v_{jk}-\bar{v_{j}}\right)}^{2}}{10\cdot 9}}$$

**Figure 7:**
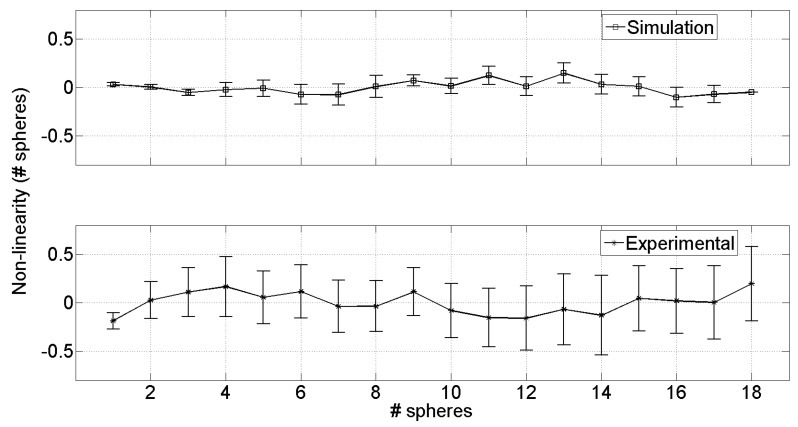
Evaluation of the non-linearity on the measurement expressed in terms of number of spheres (one sphere is 1.77 × 10^−9^ m^3^). Top graph represents the simulations, bottom graph represents experimental results. Error bars are the uncertainty expressed as standard deviation of the sample means.

Figure 7 shows the plots of linearity error for simulations (top graph) and experimental data (bottom graph); error bars represent the standard deviation of the sample means u(Lin). The results are expressed in terms of number of microspheres, and it is possible to see that both simulations and experimental results agree, showing 0.2 spheres non-linearity. This value corresponds, knowing that each sphere is 1.77 × 10^−9^ m^3^, to a non-linearity of 3.53 × 10^−10^ m^3^ (i.e., 1% of the measured volume). The agreement of non-linearity between simulated and experimental data is due to the approximation in (5); the accuracy of this approximation depends on the number of electrodes in the polygonal investigation area. With 10 voltage electrodes the non-linearity is in the order of 1%, this could be improved with a higher number of electrodes; obviously, increasing the number of electrodes means introducing more electronic components, a DAQ with more channels and longer acquisition time, thus invalidating the low-cost profile of the proposed solution.

The main difference that emerges from the two plots in Figure 7 is the uncertainty on non-linearity; the error bars in the bottom graph are, predictably, wider with respect to simulations, providing 0.4 sphere uncertainty (corresponding to 7 × 10^−10^ m^3^). This higher uncertainty is mainly due to the experimental setup, in particular to the oscillator stability and to the linearity of the current to voltage converter.

## 5. Conclusions

A technique to estimate the total volume of unknown insulating inclusions in a conducting body has been presented and experimentally validated. The technique turns out to be very cheap both in terms of hardware and software, given that no forward numerical solver solution is needed and expensive reconstruction algorithms are therefore avoided. Only a pair of driving electrodes is used that enable a simplification of the electronic hardware with respect to a similar technique proposed in literature that, in contrast, requires one independent current source for each electrode. The experimental results showed good agreement with simulations and the non-linearity of the proposed device results in the order of 1%.
